# Supplementary material for: Reliable New Biomarkers of Mitochondrial Oxidative Stress and Neuroinflammation in Cerebrospinal Fluid and Plasma from Alzheimer’s Disease Patients: A Pilot Study
Source: Int J Mol Sci. 2025 Aug 12;26(16):7792. doi: 10.3390/ijms26167792 (PMC12386766; doi:10.3390/ijms26167792)
Supplement: Supplementary file 1 [file ijms-26-07792-s001.zip › ijms-3789672-supplementary.pdf]

ORIGINAL BLOTS

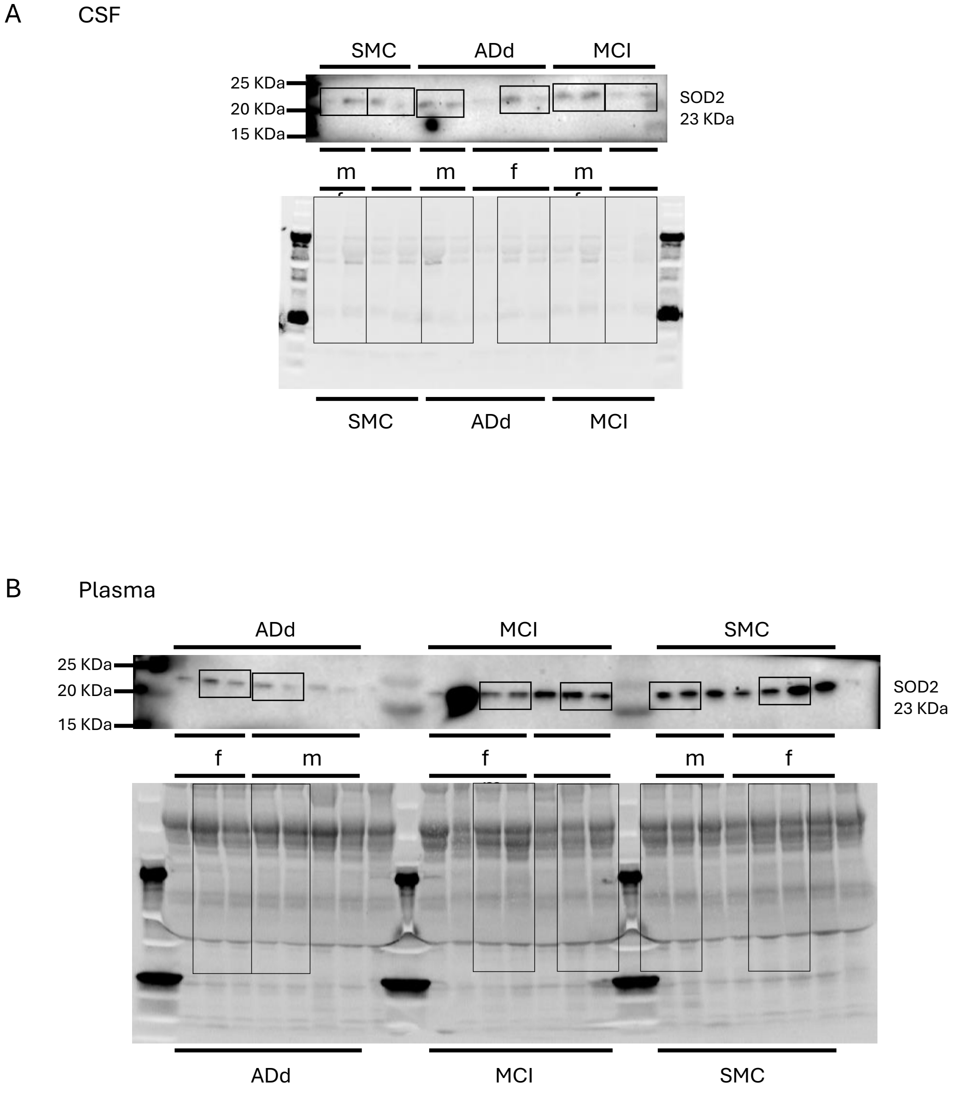

Supplementary Figure S1. Representative western blotting for SOD2 and total protein in CSF (A) and plasma (B).

Representative whole blots of SOD2 protein and total protein used in manuscript.

SMC (Subject Memory Complaint), MCI (Mild Cognitive Impairment), ADd (Alzheimer Disease dementia).

The bands enclosed in the boxes in A and in B are reported, respectively, in Figures 1G and 1H.

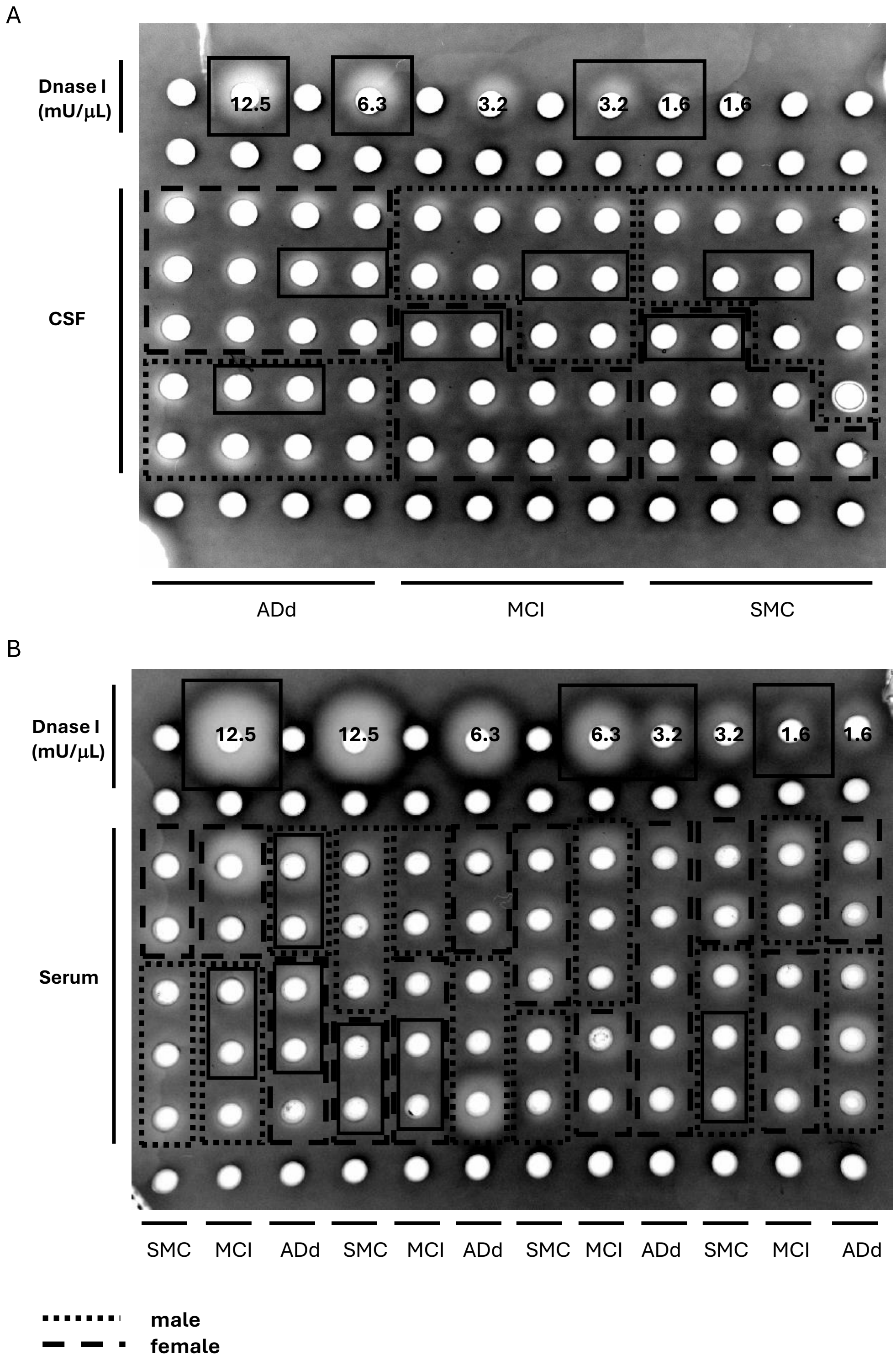

Supplementary Figure S2. Representative agarose gel for Dnase activity in CSF (A) and serum (B).
Representative agarose gel showing circular white zones, indicative of Dnase activity, obtained from single radial enzyme diffusion assay and used in manuscript.
SMC (Subject Memory Complaint), MCI (Mild Cognitive Impairment), ADd (Alzheimer Disease dementia).
Known concentrations of recombinant DNase I are loaded into the gels (A-B) as standard. 25 µL of CSF (A) and 5 µL of plasma (B) are loaded into the gel. Unmarked wells are free of sample.
The circular white zones enclosed in the boxes in A and in B are reported, respectively, in Figures 5G and 5H.
